# Benefits and detriments of unilateral cochlear implant use on bilateral auditory development in children who are deaf

**DOI:** 10.3389/fpsyg.2013.00719

**Published:** 2013-10-16

**Authors:** Karen A. Gordon, Salima Jiwani, Blake C. Papsin

**Affiliations:** ^1^Archie’s Cochlear Implant Laboratory, The Hospital for Sick ChildrenToronto, ON, Canada; ^2^Institute of Medical Sciences, Faculty of Medicine, University of TorontoToronto, ON, Canada; ^3^Department of Otolaryngology – Head and Neck surgery, Faculty of Medicine, University of TorontoToronto, ON, Canada

**Keywords:** deafness, cochlear implantation, unilateral hearing, auditory brainstem responses, auditory evoked cortical potentials, auditory development, plasticity, binaural hearing

## Abstract

We have explored both the benefits and detriments of providing electrical input through a cochlear implant in one ear to the auditory system of young children. A cochlear implant delivers electrical pulses to stimulate the auditory nerve, providing children who are deaf with access to sound. The goals of implantation are to restrict reorganization of the deprived immature auditory brain and promote development of hearing and spoken language. It is clear that limiting the duration of deprivation is a key factor. Additional considerations are the onset, etiology, and use of residual hearing as each of these can have unique effects on auditory development in the pre-implant period. New findings show that many children receiving unilateral cochlear implants are developing mature-like brainstem and thalamo-cortical responses to sound with long term use despite these sources of variability; however, there remain considerable abnormalities in cortical function. The most apparent, determined by implanting the other ear and measuring responses to acute stimulation, is a loss of normal cortical response from the deprived ear. Recent data reveal that this can be avoided in children by early implantation of both ears simultaneously or with limited delay. We conclude that auditory development requires input early in development and from both ears.

## INTRODUCTION

A cochlear implant is an auditory prosthesis which is surgically implanted into the cochlea (inner ear), and allows children who are deaf to develop oral speech and language. Because the brain is most susceptible to changes in early life, providing access to sound at a young age is essential to promote auditory development (Papsin and Gordon, 2007; Kral and O’Donoghue, 2010). The implant cannot restore normal hearing. It provides only a crude representation of acoustic sounds, eliminates important cochlear processing, and may not be able to completely reverse the effects of deafness. In addition, cochlear implants were traditionally provided unilaterally (i.e., in only one ear) in children, leaving the opposite pathways deprived of input and susceptible to degeneration and reorganization (O’Neil et al., 2010; Gordon et al., 2013; Kral et al., 2013). Yet, despite these disadvantages, many children achieve excellent listening and oral communication abilities. In the present review, we share findings from studies exploring whether cochlear implantation can limit reorganization of the deprived immature auditory brain and promote appropriate and normal-like development along the auditory pathways.

## THE AUDITORY SYSTEM REORGANIZES WHEN BILATERALLY DEPRIVED

Prior to cochlear implantation, the absence of auditory input to the auditory system leaves the brain vulnerable to reorganization (Nishimura et al., 1999; Bavelier et al., 2000, 2006; Finney et al., 2001; Lee et al., 2001; Bavelier and Neville, 2002; Merabet and Pascual-Leone, 2010). Secondary and association auditory areas, including parts of the planum temporale, all of which respond to multi-sensory input including hearing, vision and touch (Pandya and Yeterian, 1985; Giard and Peronnet, 1999; Calvert et al., 2001; Calvert and Thesen, 2004), become recruited by the visual (Finney et al., 2001; Lee et al., 2001, 2007b; Lomber et al., 2010; Meredith and Lomber, 2011) and somatosensory (Levänen et al., 1998; Levänen and Hamdorf, 2001; Auer et al., 2007; Meredith and Lomber, 2011) systems to perform non-auditory functions. As a consequence of early auditory deprivation, processing of visual peripheral localization by the posterior auditory field (Lomber et al., 2010), visual motion detection by the dorsal zone of the auditory cortex (Lomber et al., 2010), and somatosensory sensation by the anterior auditory field (Meredith and Lomber, 2011) become enhanced in individuals who are deaf. These changes appear to result from a direct competition for resources in areas which receive multi-sensory input. If governed by principals of Hebbian processing (Hebb, 1949; Abbott and Nelson, 2000; Song et al., 2000), neurons in these areas might preferentially form viable connections with non-auditory inputs to the detriment of inputs carrying auditory information. We must be concerned by the reorganization of the deaf auditory cortex because, depending on how quickly these processes occur, they may be impossible to reverse and could impair outcomes after cochlear implantation. It is also becoming clear that these changes do not occur uniformly in children who are deaf and may be related to the heterogeneity in the onset and cause of pediatric deafness (Gordon et al., 2011a,c).

Limiting the period of bilateral deafness in early life is essential to drive maturation in the auditory pathways (O’Donoghue, 1999; Kral et al., 2001; Ponton and Eggermont, 2001; Sharma et al., 2005; Papsin and Gordon, 2007; Gordon et al., 2008a, 2010; Nikolopoulos et al., 2009), and promote optimal hearing and speech and language development (Beadle et al., 2005; Harrison et al., 2005; Nicholas and Geers, 2007; Geers and Sedey, 2011). Many studies investigating auditory development after cochlear implantation focus on children who are deaf in infancy, but do not examine the larger heterogeneity in etiology, onset and/or degree of deafness. These factors may each have unique effects on auditory activity in the brain prior to implantation. For example, biallelic mutations of the Gap Junction Beta-2 (GJB-2) gene causes deficits in the cochlea at likely very early stages of development with possible consequences for auditory function after implantation (Propst et al., 2006). The GJB-2 gene normally codes for the connexin-26 protein, which creates gap junctions in the cochlea necessary for the appropriate release and maintenance of electrochemical gradients. This in turn, generates action potentials and stimulates the auditory nerve (Kelley et al., 1998; Cohn and Kelley, 1999; Gualandi et al., 2002). Electrophysiological recordings of auditory evoked cortical activity at initial cochlear implant activation in children with severe GJB-2 mutations revealed that responses from the cortex were more homogenous in this cohort compared to those children who did not have such a mutation. Auditory evoked cortical responses in children with GJB-2 mutations were characteristic of earlier stages of cortical development, perhaps reflecting restricted spontaneous activity in the auditory system and more limited access to sound prior to implantation compared to their peers who did not have a GJB-2 related deafness (Gordon et al., 2011c). This was further supported by poorer hearing sensitivity in the low frequencies in the GJB-2 group (Propst et al., 2006).

The degree of residual hearing is another important predictive factor for cochlear implant outcomes. Traditional candidacy criteria for cochlear implantation in children include a diagnosis of permanent severe-to-profound hearing loss bilaterally with little or limited access to acoustic input through hearing aids (Osberger et al., 2002). We recently reported that children who had better hearing at 250 Hz used their hearing aids for longer durations prior to receiving a cochlear implant (Hopyan et al., 2012). Of interest, these children performed significantly better on tests of music perception with their implants, particularly when detecting differences in rhythm, compared to children who did not have acoustical access to these low frequencies prior to implantation (Hopyan et al., 2012). Thus, there are advantages of acoustical input for auditory development which can be capitalized upon after cochlear implantation. In general, we are learning that the cause, onset and degree of deafness in any one child will be important to understand in order to ensure that he/she makes the best possible use of his/her device.

## UNILATERAL COCHLEAR IMPLANTATION RESTORES HEARING AND PROMOTES AUDITORY DEVELOPMENT

The cochlear implant was made available to children in North America in the early 1990s and works by stimulating the auditory pathways with electrical pulses. The implant contains an array of electrodes which is surgically placed in the scala tympani of the cochlea. These electrodes each deliver electrical pulses to stimulate the auditory nerve. External equipment is worn which takes in acoustic sound through the microphone, extracts frequency and intensity information in a speech processor and sends instructions to an internal device through an FM transmitting coil. The internal receiver-stimulator sends this information to the electrodes which are organized to mimic the normal cochlea; high frequency sounds are allocated to basal electrodes with lower frequencies being allocated to progressively more apical electrodes. In this way, the child receives an electrical representation of the acoustic world and learns to understand sounds including speech.

**FIGURE 1:**
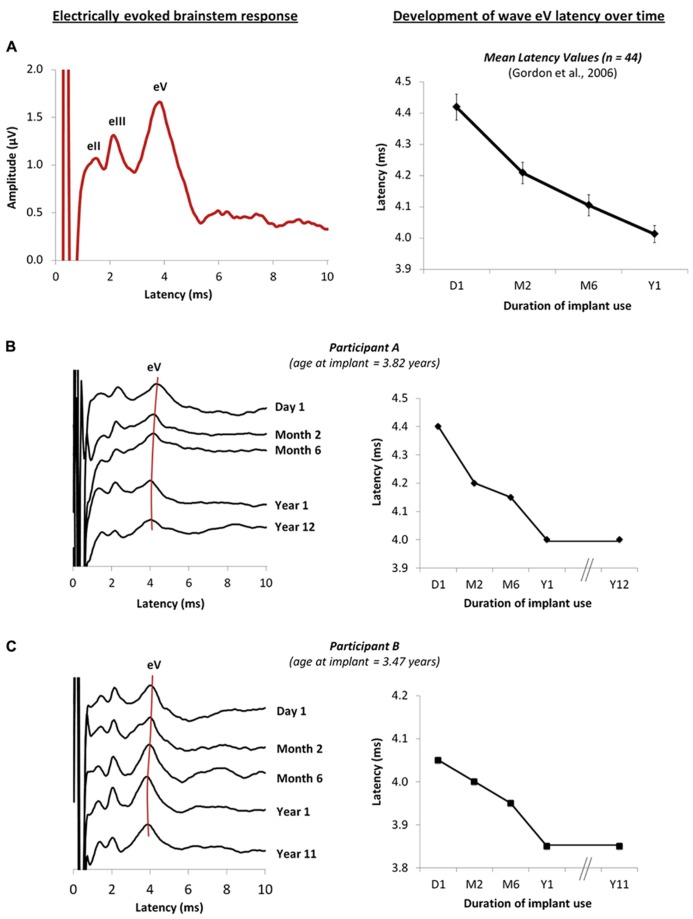
**(A)** Example of an electrically evoked auditory brainstem response waveform is shown on the left. The onset of the cochlear implant artifact is shown at time 0 ms, followed by peaks eII, eIII and eV. Data from Gordon et al. (2006) are plotted on the right and show the mean wave eV latency values of 44 children recorded at initial activation of the implant, and at months 2, 6 and 12 following unilateral cochlear implantation. **(B,C)** on the right show the changes in the brainstem responses of two children who were in the original study (Gordon et al., 2006), recorded from initial activation of the device to different intervals over the first year of cochlear implantation use. New responses recorded after 10 years of unilateral cochlear implant experience are also shown (Jiwani et al., 2011), further confirming that little change in the eV latency occurs beyond the first year of implant use. The wave eV latencies at each time-point are represented on the right for each child.

Auditory brainstem development, measured by decreasing latencies of evoked potential peaks, is largely complete by the first year of cochlear implant use in children with early onset deafness (Gordon et al., 2003, 2006), indicating increasing efficiency of neural conduction and improved neural synchrony with exposure to sound (Gordon et al., 2003). Similar changes have been reported from the auditory brainstems of normal hearing children over a similar time-course (Salamy and McKean, 1976; Starr et al., 1977; Jerger and Hall, 1980; Salamy, 1984; Hecox and Burkard, 2006). Data from Gordon et al. (2006) is shown in **Figure 1A**; on the left is an example of an electrically evoked auditory brainstem response. The stimulus artifact is shown at time 0 ms followed by waves eII, eIII and eV, and on the right, the latency values of wave eV are plotted at initial device activation and over the first year following cochlear implant use in 44 children who had early onset deafness and were implanted unilaterally (Gordon et al., 2006). Recently, we recorded these same responses in two children who were in the original study once they had over a decade of unilateral cochlear implant experience. Their responses are shown in **Figures 1B,C** (Jiwani et al., 2011). In both cases, wave eV latency clearly decreases over the first year of cochlear implant use, with no further changes thereafter. This suggests that activity in auditory brainstem is largely complete by the first year (Gordon et al., 2006).

**FIGURE 2:**
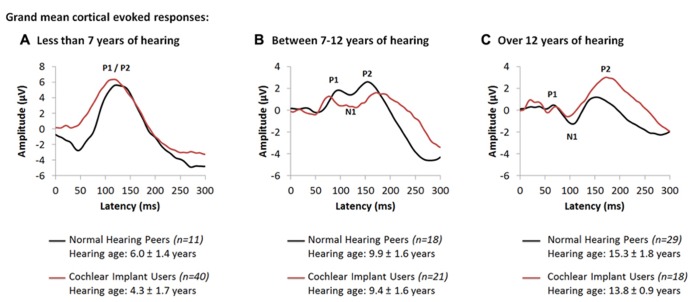
Grand mean cortical evoked responses from 79 cochlear implant users (red waveform; Jiwani et al., 2013a) are plotted for children who have **(A)** under 7 years of hearing experience (4.3 ± 1.7 years; *n* = 40), **(B)** between 7 and 12 years of hearing experience (9.4 ± 1.6 years; *n* = 21), and **(C)** those who have more than 12 years of hearing experience (13.8 ± 0.9 years; *n* = 18). Mean responses for each range of hearing experience are compared to a normal and mature cortical waveform (black waveform; Jiwani et al., 2013a), recorded from normal hearing peers who are matched for hearing age (*n* = 58).

Further studies concentrated on the development of cortical auditory activity in children with time after cochlear implantation. Cochlear implants provided to children who are congenitally deaf within 3.5 years of bilateral deafness promote age-appropriate cortical responses over the first 3–6 months of implant use (Sharma et al., 2002a). After this initial period, these responses change at a rate which is similar to normal (Eggermont et al., 1997; Eggermont and Ponton, 2003). We recently assessed changes in cortical responses after longer term unilateral cochlear implant use in children who were implanted early (Jiwani et al., 2013a). Grand mean cortical evoked responses from 79 unilateral cochlear implant users (red waveforms) are plotted in **Figure 2** along with the grand mean responses from 58 normal hearing peers (black waveforms) for different intervals of hearing experience. **Figures 2A–C** show grand mean cortical evoked waveforms from children who have between 0 and 7 years (40 cochlear implant users; 11 normal hearing), 7 to 12 years (21 cochlear implant users; 18 normal hearing) and over 12 years (18 cochlear implant users; 29 normal hearing) of hearing experience, respectively. Cochlear implant users represented in these Figures had limited durations of bilateral deafness prior to implantation (2.03 ± 1.36 years) with typical heterogeneity in their etiologies of deafness.

As shown in **Figure 2A**, responses from children with up to 7 years of hearing experience with an implant or with normal bilateral hearing are dominated by a large and broad positive amplitude peak, labeled P_1_/P_2_. Comparison of peak latencies (*t*_(__47.3__)_ = -1.63; *p* > 0.05) and amplitudes (*t*_(__42.1__)_ = -0.64; *p* > 0.05) reveal no significant differences between the two groups. This positive-peaked response is believed to reflect either excitatory auditory activity from the thalamus to deep layers of the auditory cortex (Liegeois-Chauvel et al., 1994), or auditory driven activity from association auditory areas to the reticular activating system in the non-lemniscal auditory pathways (Kraus et al., 1992; Ponton et al., 2000; Ponton and Eggermont, 2001). As thalamo-cortical and cortico-cortical connections develop around 9 to 12 years of age in superficial layers of the auditory cortex, a small negative amplitude peak, labeled N_1_, develops in the cortical evoked response and bifurcates the large P_1_/P_2_ response into three peaks: P_1_, N_1_ and P_2_. Similar developmental changes to the cortical response are observed in early implanted cochlear implant users who have equal durations of hearing experience. Indeed, as shown in **Figure 2B**, with 7 to 12 years of auditory experience (9.38 ± 1.57 years in cochlear implant users; 9.92 ± 1.57 years in normal hearing individuals), the cortical response in both groups begins to develop into a polyphasic waveform. The grand mean response from all 21 unilaterally implanted children begins to bifurcate into a 3-peaked cortical response at this stage of implant use (**Figure 2B**). Differences in the wavepeak latencies (P_1_: *t*_(10)_ = -0.88, *p* > 0.05; N_1_: *t*_(10.18)_ = -1.3, *p* > 0.05; P_2_: *t*_(10.77) _= 1.43, *p* > 0.05) and peak-to-peak amplitudes (P_1_-N_1_: *t*_(6.87)_ = 1.75, *p* > 0.05; N_1_-P_2_: *t*_(10.67)_ = 2.2, *p* > 0.05) between both groups were not significant. This response continues to develop with time. As auditory pathways mature in the auditory cortex, peaks P_1_-N_1_-P_2_-N_2_ become clearly present (**Figure 2C**) when auditory experience exceeds 12 years in all 18 cochlear implant users (13.81 ± 0.92 years of unilateral implant experience) and 29 normal hearing peers (15.30 ± 1.81 years of age and hearing) (Jiwani et al., 2013a).

The data from individuals with normal hearing shown in **Figure 2** is consistent with findings by Ponton, Eggermont and colleagues who suggested that peak N_1_ normally emerges around 9 to 12 years of age reflecting maturation of thalamo-cortical and cortico-cortical loops in superficial layers of the auditory cortex (Ponton et al., 2000; Eggermont and Ponton, 2003). These pathways mediate the transfer of primary auditory and multi-sensory input from the thalamus to various regions of the ipsilateral and contralateral auditory cortices (Winer et al., 2001, 2005; Razak et al., 2009), and the transmission of information from the auditory cortex to primary and secondary sensory areas in both hemispheres (Read et al., 2002; Lee and Winer, 2005; Klinge et al., 2010). The developmental trajectory of the electrically evoked cortical waveform suggests that similar development is taking place in children using cochlear implant (Jiwani et al., 2013a), perhaps establishing: (1) appropriate relay of auditory input from the ear to the cortex, via the thalamus; (2) communication between the two cortical hemispheres; and/or (3) connectivity between different sensory areas (Jiwani et al., 2013a). These normal-like developmental changes to the auditory cortex may underlie the impressive improvements in auditory function observed with cochlear implant use over time (Beadle et al., 2005; Nicholas and Geers, 2007; Geers and Sedey, 2011).

## DIFFERENCES FROM NORMAL PERSIST IN AUDITORY PROCESSING DESPITE LONG DURATIONS OF UNILATERAL COCHLEAR IMPLANT USE

Although early implantation of young children results in normal-like cortical response peaks, as shown in **Figure 2C**, the waveform has at least one abnormality. Specifically, the amplitude of the P_2_ peak in cochlear implant users is larger than in normal hearing peers (*t*_(14.51)_ = 2.49, *p* < 0.05) (Jiwani et al., 2013a). The importance of this recent finding is that it suggests that deviations from normal cortical processing remain in these young people despite long-term unilateral implant use. Enhanced P_2_ peak amplitudes in normal hearing adults are known to reflect increases in selective attention (Picton and Hillyard, 1974; Hocherman et al., 1976; Rif et al., 1991; García-Larrea et al., 1992; Posner and Dehaene, 1994; Grady et al., 1997; Fujiwara et al., 1998; Tremblay et al., 2009) and increases in multi-sensory integration during auditory processing (Hari, 1990; García-Larrea et al., 1992; Levänen et al., 1998; Webster and Colrain, 2000; Moller and Rollins, 2002; Crowley and Colrain, 2004; Johnson and Zatorre, 2005). These processes cause a reduction in the primary network which becomes supplemented by the frontal and parietal areas through increased neural recruitment and synchrony (Tremblay et al., 2001, 2009; Tremblay and Kraus, 2002; Tremblay, 2007) from the non-primary and association auditory pathways (Hocherman et al., 1976; Kraus and McGee, 1993; Kraus et al., 1994; Grady et al., 1997; Busse et al., 2005). It is therefore possible that the larger than normal amplitude of peak P_2_ observed in children with long-term cochlear implant experience reflects increased cognitive demands for attention and multi-sensory system integration during hearing (Jiwani et al., 2013a). This may reflect compensatory mechanisms to offset: (1) the reorganization in the auditory brain potentially occurring during the period of deafness prior to implantation; (2) the abnormal auditory input provided by the cochlear implant; and/or, (3) the absence of sound to the un-implanted ear which may lead to reorganization in the deprived pathways.

Cochlear implant users compensate for the abnormal input they receive through the device (Doucet et al., 2006; Giraud and Lee, 2007; Lee et al., 2007a,b; Hopyan-Misakyan et al., 2009; Strelnikov et al., 2010; Hopyan et al., 2011; Kral and Sharma, 2011; Lazard et al., 2011, 2012; Hopyan et al., 2012; Sandmann et al., 2012). We found that children using cochlear implants depend on visual cues more heavily than normal to listen for complex information embedded in speech (Hopyan-Misakyan et al., 2009). Emotion perception was tested using 2 subtests of the standardized Diagnostic Analysis of Nonverbal Behavior-2 (DANVA-2) in 18 cochlear implant users who received one implant by 2.9 ± 0.9 years, had 7.2 ± 1.3 years of cochlear implant experience at the time of the test, and had good speech perception skills. In the first test, children listened to the spoken sentence: “I’m going out of the room now and I’ll be back later” (24 trials), and had to decide which 1 of 4 emotions (happy, sad, angry or fearful) was conveyed by the voice. In the second test, children watched pictures of other children’s faces, each depicting one of the same four emotions, and had to decide which emotion was conveyed by the photographs. Performance accuracy was assessed for each task, and compared to 18 normal hearing controls who were matched for age (10.3 ± 1.5 years of age) (Hopyan-Misakyan et al., 2009).

Children using cochlear implants showed significantly poorer than normal performance on the emotion identification task in the auditory subtest (*F*(1,34) = 43.7, *p* > 0.01). This deficit does not reflect a general failure to identify emotions, however, since they performed as well as their peers with normal hearing when the emotions were presented in the visual modality (*F*(1,34) = 0.1, *p* > 0.05) (Hopyan-Misakyan et al., 2009). The inability of these children to perceive emotions in speech might reflect abnormal development of cortical representation of emotional prosody in speech without normal hearing (Nishimura et al., 1999; Lee et al., 2001, 2007b; Doucet et al., 2006; Meredith and Lomber, 2011; Sandmann, 2012; Sandmann et al., 2012).

In sum, unilateral cochlear implantation promotes the development of normal-like activity in the auditory pathways over the long-term, but functional abnormalities persist. These could reflect: (1) deleterious or irreversible changes to neural reorganization which occurred during the period of auditory deprivation in early life, (2) abnormal representation of sound through electrical pulses stimulation of the auditory system, and/or (3) abnormal cortical development driven by the absence of auditory input to the deprived pathways from the opposite un-implanted ear. We have been studying effects of the latter issue in children.

## BINAURAL HEARING IS NOT AVAILABLE TO TRADITIONAL UNILATERAL COCHLEAR IMPLANT USERS

Hearing through only one cochlear implant eliminates access to binaural hearing, which is the ability of the auditory system to process and integrate auditory input from both ears. Binaural hearing is especially important for children because they are rarely in one place and listening to a single speaker at a time. Children need to attend to and discriminate between several sound sources when playing and learning. The noise, reverberation and distance, predominant in most situations including typical classrooms, make it challenging for children to listen and learn when binaural cues are not accessible. For children who are deaf in both ears, binaural hearing might be achieved with bilateral cochlear implantation (i.e., cochlear implants in both ears) (van Hoesel and Tyler, 2003; Litovsky et al., 2004, 2006; Brown and Balkany, 2007; Steffens et al., 2008b; Basura et al., 2009; Eapen and Buchman, 2009; Gordon et al., 2010, 2011b; Salloum et al., 2010; Chadha et al., 2011). Bilateral cochlear implantation is now being increasingly provided to children either in the same surgery (simultaneously) or in two different surgeries following a period of unilateral implant use (sequentially).

Bilateral cochlear implants attempt to restore binaural hearing by providing information to both ears. Normally, the auditory system compares, processes and integrates subtle differences between level and timing of sounds reaching each ear. In this way, binaural hearing allows: (1) the identification/localization of sound sources in space (Batteau, 1967; Lorenzi et al., 1999; Van Deun et al., 2009b; Grothe et al., 2010); (2) increased perception of loudness through binaural summation (Bocca, 1955; Blegvad, 1975); and (3) improved hearing in quiet and in noisy environments through the head shadow and squelch effects (Hawley et al., 2004; Van Wanrooij and Van Opstal, 2004). Binaural hearing also makes communication less tiring which enables listening and communication to be a more pleasant experience. Although restoring binaural hearing is the goal of bilateral implantation, this has not been completely realized in either adults or children (van Hoesel and Tyler, 2003; Seeber and Fastl, 2008; Grieco-Calub and Litovsky, 2010; Salloum et al., 2010).

Children who are deaf in both ears hear speech better with bilateral cochlear implants than unilateral implants (Litovsky et al., 2004; Brown and Balkany, 2007; Ching et al., 2007; Galvin et al., 2007; Peters et al., 2007; Seeber and Fastl, 2008; Steffens et al., 2008a; Basura et al., 2009; Eapen and Buchman, 2009; Gordon and Papsin, 2009; Van Deun et al., 2009a; Salloum et al., 2010; Chadha et al., 2011), but do not hear binaural cues normally (Grieco-Calub and Litovsky, 2010; Salloum et al., 2010). Outcomes improve when both implants are provided with limited delays and at young ages (van Hoesel and Tyler, 2003; Gordon and Papsin, 2009; Van Deun et al., 2009a; Gordon et al., 2010; Chadha et al., 2011). As the duration of inter-implant delay decreases, the two ears develop more symmetric speech perception abilities and children show increasing advantages of bilateral over unilateral implantation (Gordon and Papsin, 2009). Significant improvements on standardized speech perception tests are seen as early as 6 months following bilateral cochlear implant stimulation in children who receive their second implant simultaneously or within short delays (Gordon and Papsin, 2009). Furthermore, children implanted with both cochlear implants simultaneously derive significantly more benefit from spatial separation of noise compared to children who have longer delays between implants (Chadha et al., 2011). Sound localization improves in children who are provided access to sound early and in both ears (Van Deun et al., 2009a). By contrast, children who receive both cochlear implants sequentially after long inter-implant delays (>2 years) have persistent asymmetries in auditory function and compromised bilateral benefits for speech perception, even after 36 months of bilateral cochlear implant use (Gordon and Papsin, 2009). Sequentially implanted children also seem to depend more on their first implanted ear than their second for speech perception, and show less bilateral improvement (relative to unilateral implant use) on speech outcomes than children implanted simultaneously or with limited delay (Gordon and Papsin, 2009). These children localize sound inaccurately and rely heavily on level cues to do so (Grieco-Calub and Litovsky, 2010). The negative effect of inter-implant delay might be explained by underlying changes to the developing auditory pathways before and after unilateral and bilateral implantation.

## EVIDENCE OF A SHORT SENSITIVE PERIOD FOR BILATERAL INPUT IN HUMAN AUDITORY DEVELOPMENT

Data presented in **Figures 1 and 2** show that unilateral stimulation promotes development of the auditory pathways (Jiwani et al., 2013a), thus limiting effects of deafness. At the same time, this development might occur at the expense of pathways from the opposite and deprived ear. This might be explained by the absence of inhibition which would normally have come from input from the opposite ear during binaural hearing (Grothe et al., 2010). Without this inhibition, ascending projections from the stimulated ear may be abnormally strengthened in children who are deaf and use unilateral cochlear implants.

We studied bilateral auditory function in children who had different durations of unilateral exposure. We hypothesized that the stage of unilaterally driven brainstem development would be an important factor to consider. Perhaps changes occurring in the brainstem at earlier stages of unilaterally driven development would have less long lasting consequences on the bilateral pathways than after the unilaterally stimulated brainstem reached maturity. Development in the auditory brainstem is largely complete by 1 year of unilateral implant use (Gordon et al., 2006). Thus, children with >2 years of unilateral experience were categorized as having mature auditory brainstem function and long unilateral use. Children with <1 year of unilateral experience were considered to have short-term use with continuing auditory brainstem development. Auditory development in these children was compared to that of children who were deaf and had not yet used cochlear implants (i.e., limited to no auditory brainstem development). All children were implanted bilaterally, allowing us to assess auditory brainstem function evoked by stimulation from each ear. All children receiving bilateral implants sequentially showed brainstem responses which were faster when evoked by the experienced ear compared to the newly implanted ear at initial bilateral implant use (Gordon et al., 2008b). This was expected and confirmed earlier findings that the first implant promoted improved neural conduction through the brainstem. Repeated tests completed after 1.7 ± 1.65 year of bilateral implant use indicated that mismatches in response latencies persisted in a group of children receiving the second implant after a long delay (>2 years) (Gordon et al., 2012). Increased response latencies in response to sound from the second implanted side could reflect decreased axonal myelination, longer neural conduction times, slower or weaker synapses or more asynchronous neural activity – all signs of more limited brainstem development. Abnormal mismatches between brainstem response latencies were never present in children receiving bilateral implants simultaneously and resolved with bilateral implant use in children who received both implants after a short inter-implant delay (<1 year) (Gordon et al., 2007, 2008b, 2011b, 2012). Thus, allowing the brainstem to develop unilaterally for >2 years compromises the later promotion of symmetrically functioning bilateral auditory brainstem pathways.

**FIGURE 3:**
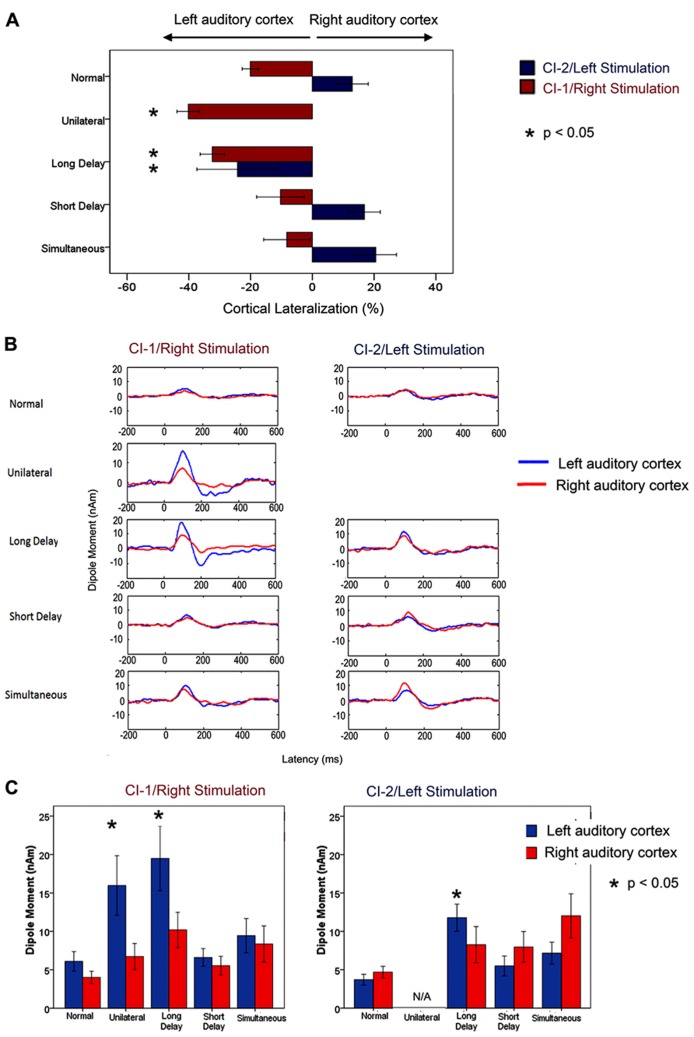
**Re-printed with permission from Gordon et al. (2013).** “**(A)** Per cent cortical lateralization (mean ± 1 standard error) is plotted for each participant group. Greater than normal contralateral lateralization to right/CI-1 stimuli was found in long delay and unilateral cochlear implant users (*p* < 0.05 and <0.0001, respectively) but not in short delay and simultaneous groups (*p* > 0.05). The long delay group showed a decrease in contralateral lateralization/increase in ipsilateral lateralization relative to those with normal hearing in response to left/CI-2 stimulation. This did not occur in the short delay and simultaneous groups. **(B)** Grand mean virtual sensor data for left and right hemispheric sources of P1 (normal hearing) and P1ci (cochlear implant users for stimulation from right/CI-1 and left/CI-2). Large peaks in responses to CI-1 (right) stimulation can be seen in the long delay and unilateral group data. **(C)** Left and right hemispheric dipole moments (mean ± 1 SE) for P1/P1ci in each group in response to right/CI-1 and left/CI-2 stimulation. In response to CI-1 (right) stimulation, there is a marked increase in left hemispheric dipole moments in participant groups with >2 years of unilateral hearing experience (long delay and unilateral; *p* < 0.05).” (Gordon et al., 2013; Brain, Figure 7, p. 11)

Mismatched bilateral auditory development in sequentially implanted children was not restricted to the brainstem. Effects of asymmetric activity in the pathways from the first stimulated ear were also found in the auditory cortex. Consistent with the brainstem findings, cortical abnormalities were not resolved by chronic bilateral implant use (3.57 ± 0.74 years) when unilateral experience exceeded 1.5 years in children who were implanted early (1.87 ± 1.25 years of age). These findings were recently reported by Gordon et al. (2013) and are shown in **Figure 3** (re-printed from that paper). We used a unique and validated “Time Restricted Artifact and Coherent Suppression” (TRACS) beamformer method (Wong and Gordon, 2009) to suppress the electrical artifact from the cochlear implant device and spatially localize areas of cortical activity in hemispheres ipsilateral and contralateral to stimulation. Like many imaging methods, the brain was divided into thousands of 3-dimensional coordinate spaces (voxels). Responses were recorded at 64-cephalic surface electrodes and the contribution of the dipole centered in each voxel to the measured field was assessed by the adaptive spatial filter of the TRACS beamformer. Dipole moments for a given voxel were calculated across latency (virtual sensor) and peak values were used for analyses.

Cortical responses were evoked by unilateral electrical pulse trains delivered from one implant electrode in seven children with normal hearing, eight children who were implanted unilaterally in the right ear (2.32 ± 1.61 years) and had 7.21 ± 2.48 years of hearing experience and 26 children who used bilateral cochlear implants for 3.42 ± 0.59 years. Of the bilateral implant users, 10 children received both cochlear implants simultaneously and 16 were sequentially implanted (right ear implanted first with no hearing aid in the left ear). Bilateral deafness prior to implantation was limited (1.74 ± 0.90 years) in all children. The children in this study had less than 12 years of hearing experience, and therefore all produced a cortical evoked response which was dominated by an immature large amplitude positive peak, similar to the one shown in **Figure 2A**. The differences between the dipoles from the left and right auditory cortices were normalized as a percent lateralization [% lateralization = (dipole right - dipole left)/(dipole right + dipole left) × 100].

A larger than normal variability in the lateralization of cortical dipoles was found in children receiving bilateral cochlear implants sequentially. A factor analysis of multiple demographic variables identified the duration of unilateral implant use as the factor which best accounted for the spread of cortical responses. We thus further analyzed the cortical lateralization data for effects of duration of unilateral implant use occurring prior to bilateral implantation. When responses were evoked by the first (i.e., right) implant, there was an increase in lateralization of activity to the contralateral left auditory cortex with unilateral implant use. This became significantly larger than the percent of cortical lateralization in the simultaneously implanted group at 1.48 years of unilateral implant use. Consistent results were obtained in data evoked by the second (i.e., left) implant but, in this case, cortical lateralization changed from the normally expected contralateral direction to ipsilateral lateralization with unilateral implant use. This abnormal switch to larger activity in the ipsilateral auditory cortex became significantly different from responses in the simultaneously implanted group by 1.37 years of unilateral implant use. These analyses indicated that children with longer than approximately 1.5 years of unilateral implant use had experienced an abnormal strengthening of pathways from their first implanted right ear through the auditory brainstem (Gordon et al., 2008b, 2012) to their left contralateral cortex. This was not resolved by several years of bilateral implant use and was associated with poorer speech perception in the second than first implanted ear (Gordon et al., 2013).

The importance of restricting unilateral implant use to less than 1.5 years is further evident in **Figure 3** (reprinted from Gordon et al., 2013). Here, the grand mean lateralization of cortical activity are shown (**Figure 3A**), as well as the grand mean dipole moments identified from the virtual sensors in each hemisphere (**Figure 3B**). The group of 16 sequentially implanted children have been divided into two groups based on the cut off of 1.5 years of unilateral implant use. The Short Delay group includes seven children who had 0.86 ± 0.1 years of unilateral implant experience at the time of testing. The other nine children, the Long Delay group, had more than 2 years of unilateral implant use (3.44 ± 1.27 years). The single positive peaked response is clear in all of the group averaged waveforms shown in **Figure 3B**. The maximum dipoles were marked and analyzed in each child. The left plot of **Figure 3C** shows that dipoles evoked by stimulation from the first/right implanted ear resulted in significantly higher dipoles in the left auditory cortex (blue bars) of children who had >1.5 years of unilateral implant use (Unilateral and Long Delay groups) than other groups of children (*F*(4,36) = 3.52, *p* < 0.05). The similar findings for these two groups confirm that unilaterally driven strengthening of projections to the contralateral left auditory cortex was not reversed by the addition of a second cochlear implant. This was true despite the children in the Long Delay group having had several years of bilateral implant experience at the time of the test. The right plot in **Figure 3C** shows mean dipoles for each auditory cortex in response to left/second cochlear implant stimulation. The Long Delay group shows significantly higher dipole moments in the left auditory cortex than the other groups of children (*F*(3,29) = 5.31, *p* < 0.01). Thus, regardless of which ear was stimulated, the left auditory cortex (contralateral to the first/right implanted ear) was the more active side of the brain in children who had used one implant for >1.5 years. One explanation for this finding is that the specialized processing of language in left auditory cortex (Zatorre and Belin, 2001; Zatorre et al., 2002; Tervaniemi and Hugdahl, 2003; Firszt et al., 2006) is abnormally increased in unilateral cochlear implant users. It is not clear, however, how such a network would have been recruited by the simple non-speech stimuli used in the present experiment. An alternate explanation is that unilateral stimulation allowed abnormal strengthening of pathways from that ear.

Further evidence that the cortical changes were due to unilaterally driven strengthening was found by assessing activity in the ipsilateral/right auditory cortex. We assessed which ear preferentially activated the hemisphere contralateral to the ear deprived during the period of unilateral implant use (i.e., the right auditory cortex). The right auditory cortex was expected to respond more strongly to input from the left than right ear because the majority of neurons from one ear normally cross to the contralateral brainstem and ascend ipsilaterally from there. This was confirmed in the group of children with normal hearing and children with limited unilateral implant use prior to bilateral implantation (short delay and simultaneous). By contrast, this pattern was reversed in children in the Long Delay group. This meant that this group of children had experienced a strengthening of pathways from their hearing ear to both the ipsilateral (right) cortex, as shown by the reversal of aural preference, as well as the contralateral (left) cortex as shown by the data in **Figure 3**. The same reversal of aural preference in the cortex ipsilateral to the hearing ear has recently been reported in congenitally deaf white cats (Kral et al., 2013).

The abnormal strengthening of pathways from the unilaterally hearing ear to the immature brain seems to initially occur at the level of the brainstem. This is supported by evidence of mismatched brainstem latencies observed from children with long (>2 years) unilateral hearing experience (Gordon et al., 2012). The shorter wave eV latencies evoked from the more experienced ear suggest an increasing efficiency of activity from this side and a weakening of pathways from the opposite ear, as reflected by slower peak latencies on the second implanted side. This could result from a lack of inhibitory processes in the brainstem which are normally present during binaural hearing (Grothe et al., 2010). Listening from one side would allow auditory input from the first right implanted side to be projected to the cortex with abnormally high excitation during development thus strengthening pathways to the contralateral cortex. It appears that if this is allowed to occur until the brainstem is largely developed (i.e., >1 year of unilateral implant use), it establishes asymmetric activity in the auditory pathways which is not easily reversed by providing a second implant in the deprived ear. Limiting the period of unilateral hearing in children by providing bilateral cochlear implants with little or no delay appears to protect the bilateral pathways from this abnormal development. These findings thus suggest that there is a sensitive period of 1.5 years for binaural auditory development in children.

## LONG-TERM UNILATERAL IMPLANT USE IN OLDER CHILDREN CAUSES LASTING ASYMMETRY IN THE BILATERAL AUDITORY PATHWAYS

We make the case above that unilateral implant use in children who have been deaf since infancy should be limited to less than 1.5 years to promote normal-like symmetrical development of the auditory pathways from both ears. However, providing bilateral implants within this time frame may not always be possible. For example, many adolescents/young adults who were implanted as babies and have already had many years of unilateral hearing experience are now seeking a cochlear implant for their opposite ear in hopes of deriving benefits of bilateral implantation. These children are different in several ways from our previous research cohorts of sequentially implanted children. They have had very long periods of unilateral cochlear implant use concurrently with long durations of deprivation in their non-implanted ear, and they are no longer children. We thus expect unique cortical development in this new group of bilateral implant users, relative to our previous study groups.

**FIGURE 4:**
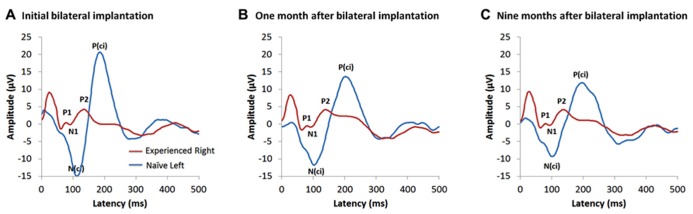
**Example of cortical evoked responses from an adolescent in the Jiwani et al. (2013b, c) study cohorts.** She received a right unilateral cochlear implant (red waveform) within limited durations of bilateral deafness (3 years of age) and used it unilaterally to hear for 15.95 years. She then received a second implant in the opposite and deprived left side (naïve side; blue waveform). Cortical responses evoked from both implants are shown at: **(A)** the first day of activation of the second implanted ear (Jiwani et al., 2013b), **(B)** one month after bilateral implantation (Jiwani et al., 2013c) and **(C)** 9 months following bilateral cochlear implant experience (Jiwani et al., 2013c).

**Figure 4** shows the cortical responses recorded at a midline cephalic location on the head (Cz) and evoked by cochlear implant stimulation from each ear on the first day of activation of the second implant in a child who had 15.95 years of hearing experience on the right side and was deprived of auditory input in the left ear. These measures were repeated after 1 month of bilateral implant use and then again after 9 months (Jiwani et al., 2013b,c). Responses from the latter two time points are shown in **Figures 4BC**, respectively. The red waveform shows the grand mean response recorded from the side with long-term unilateral cochlear implant experience, and the blue is the cortical waveform evoked by stimulation of the newly implanted side (naïve side). The two responses are very different from one another at all time points. Consistent with previous findings, the cortical responses from the experienced side (red waveform) in **Figure 4** were dominated by a mature-like morphology, comprised of the obligatory peaks P_1_-N_1_-P_2_-N_2_, similar to those expected in same aged peers with normal hearing (Jiwani et al., 2013a). By contrast, responses recorded from the newly implanted ear (blue waveform) were characterized by different peaks occurring with much larger amplitudes than the responses from the side with long-term hearing experience; a large negative peak (*N*_(ci)_), followed by a large positive peak (*P*_(ci)_) can be seen (Jiwani et al., 2013b,c). Little changes to either response occurred over the first months of bilateral implant use. Slight decreases in the latencies and amplitudes of the peaks evoked by the newly implanted ear were found after one month (**Figure 4B**), with almost no change in latency, amplitude or waveform morphology thereafter. This is shown by the response recorded at 9 months following activation of the second implant in **Figure 4C** (Jiwani et al., 2013c).

The lack of cortical development evoked by stimulation of the second implanted side is in contrast to the rapid developmental change expected to occur at early stages of unilateral cochlear implant use in young children (Sharma et al., 2002a; Sharma and Dorman, 2006), and, rather, more similar to the limited change reported in older children implanted after long durations of bilateral deafness (Sharma et al., 2002b; Gordon et al., 2005, 2008a). This might reflect immaturity or abnormalities in auditory development from the second implanted side, driven by either long duration of auditory deprivation or by maturation of the auditory cortex from unilateral cochlear implant use. Providing a second implant to children after this period has passed may prevent the naïve cortical pathways from developing after an important period in cortical auditory development has been missed. The findings from our previous study (Gordon et al., 2013) (discussed above and shown in **Figure 3**) suggest that there is an early sensitive period for bilateral brainstem development (exceeded after 1.5 years of unilateral implant use) and a later cortical maturation promoted by unilateral use of over 10 years (Jiwani et al., 2013a), as shown by the data in **Figure 4** (Jiwani et al., 2013b, c). Together, these results suggest that there are multiple sensitive periods in the developing auditory system.

## BILATERAL IMPLANTATION WITHIN A SENSITIVE PERIOD IMPROVES PERCEPTION OF BINAURAL TIMING CUES

As reviewed above, several lines of investigation suggest that the potential for promoting binaural hearing in children who are deaf will be best realized by limiting the period of bilateral deafness and providing bilateral implants with little delay. We have been studying the perception of binaural level and timing cues in children who received bilateral cochlear implants because these cues are important for binaural hearing. Interaural level and timing cues arise because sounds coming from one side of the head reach the closer ear at higher intensities and/or faster than the other ear. Level and timing differences are coded in the auditory brainstem by the degree of inhibition (Grothe et al., 2010).

We found that 19 children receiving one implant at 2.1 ± 1.1 years of age and the second after 4.9 ± 2.8 years of unilateral implant use can hear changes in interaural level differences but have particularly poor abilities to detect interaural timing cues even after several years of bilateral cochlear implant use (Salloum et al., 2010). Poor detection of binaural timing cues by sequentially implanted children was surprising given evidence from a similar group showing that the auditory brainstem integrates input from both implants as measured by the electrophysiological binaural interaction component (Gordon et al., 2012). This measure is a calculated difference between the sum of the left and right evoked auditory brainstem responses and the bilaterally evoked brainstem response. Peaks in the difference response reflect inhibition occurring with binaural processing (Dobie and Berlin, 1979; Dobie and Norton, 1980; Brantberg et al., 1999). Using this difference measure, we found that tonotopic organization is maintained in the bilateral brainstem of children who are deaf and that the pathways continue to code interaural level cues despite development driven from one ear before the other. There are consequences of the mismatches in development resulting from unilateral implant use. Although the auditory brainstem codes interaural timing differences, this does not occur normally (Gordon et al., 2008b). A miscalculation of binaural brainstem interactions results from the mismatch in neural conduction (measured by shorter peak latencies responses from the more experienced ear). More recent findings show that a sound arriving first to the more experienced ear by 1ms, for example, reduces the binaural interaural component more than when it arrives first by the same amount to the second implanted ear (Gordon, et al., in preparation). Nonetheless, coding of interaural timing remains (albeit abnormally calibrated); thus abnormal brainstem processing cannot account for the profound difficulties these children have detecting timing differences sent by their bilateral implants. This suggests a deficit for interaural timing processing in more central areas of the auditory system which likely occurred during the period before bilateral implantation. In support, the numbers of cortical neurons specialized to respond to interaural timing cues are reduced in congenitally deaf white cats (Tillein et al., 2010) as are numbers of neurons in auditory cortices responsible for sound localization (Malhotra et al., 2008). In more recent work, we are asking whether binaural timing cues are better heard by children who received bilateral cochlear implants simultaneously. Preliminary findings suggest good potential for development of binaural hearing in children who have limited durations of bilateral and unilateral deafness, but is compromised in children with long unilateral cochlear implants experience (>1.5 years).

## CONCLUSION

We have reviewed evidence showing that access to sound within limited durations of bilateral deafness in early life promotes normal-like development of activity along the auditory pathways in children who have many years of hearing experience with a unilateral cochlear implant. At the same time, however, the unilaterally driven stimulation leaves the opposite pathways deprived of input and susceptible to reorganization. We find that providing bilateral cochlear implants to children after a period of unilateral deafness of longer than 1.5 years drives abnormal mismatches in activity at the level of the brainstem and cortex. This is characterized by abnormal strengthening of activity to both the contralateral and ipsilateral auditory cortices from the first implanted ear. These abnormalities in auditory development are associated with more asymmetric speech perception, poorer hearing in noise, abnormal sound localization, and an inability to identify inter-aural timing cues. These skills are important for normal integration and processing of auditory input. We therefore suggest that binaural hearing is compromised in children who receive bilateral cochlear implants after a period of unilateral implant use exceeding 1.5 years. With that in mind, cochlear implants should be provided to children early as well as bilaterally within very limited or no delays between implants (i.e., simultaneously). Our current studies are now examining how much residual hearing is needed in the un-implanted ear to provide a potential protective effect against unilaterally driven reorganization and whether bimodal hearing (acoustic and electrical input) can be used to restore binaural hearing. Further, we are asking whether the sensitive period for bilateral input can be “reopened” by attempting to strengthen pathways from the second implanted ear to restore symmetric bilateral pathways and binaural hearing. Our findings suggest that both bilateral and unilateral deprivation should be limited to promote optimal binaural hearing in children who use cochlear implants, and enable them to function better and more naturally in challenging listening situations such as the playground or classroom environments.

## Conflict of Interest Statement

The authors declare that the research was conducted in the absence of any commercial or financial relationships that could be construed as a potential conflict of interest.
